# Amplifying missing voices in healthcare research: an AI framework for co-production of PPIE

**DOI:** 10.3389/fdgth.2026.1771729

**Published:** 2026-04-07

**Authors:** Andrew Steele, Fiona Strachan, Christine Milligan, Tracy Ibbotson

**Affiliations:** 1 College of Medical, Veterinary & Life Sciences, University of Glasgow, Glasgow, Scotland; 2 NHS Golden Jubilee University National Hospital, Clydebank, Scotland; 3 Usher Institute, University of Edinburgh, Edinburgh, Scotland; 4 School of Health & Wellbeing, University of Glasgow, Glasgow, Scotland

**Keywords:** co-production, digital health, health equity, large language model, natural language processing, patient and public involvement and engagement, synthetic personas, text analytics

## Abstract

Patient and Public Involvement and Engagement (PPIE) is essential for high-quality healthcare research, yet significant challenges persist in achieving diverse input. Traditional PPIE panels can struggle with recruitment limitations, geographical constraints, and resource intensity, resulting in panels that may not reflect population diversity or lived experiences. In order to address these challenges, we developed Panelyze, an AI-powered co-production system to augment existing PPIE approaches. Panelyze follows a five-step workflow: (1) programmatic generation of synthetic personas and panels based on census data and lived experiences; (2) semantic analysis of research proposals; (3) generation of the synthetic panel's discussions; (4) generation of PPIE co-production artefacts (e.g., Plain English Summaries, Infographics); and (5) generation of the Panelyze Score, a novel algorithmic metric evaluating healthcare proposals against UK Standards for Public Involvement. We validated the system using the CAPRIE-2 cardiovascular research proposal. Results demonstrated the system's capacity to simulate qualitative dialogue, identifying critical socio-economic barriers (e.g., wage loss from clinic visits) and validating terminology choices (e.g., drug naming conventions). Critically, the system functions as an augmentation tool, enabling research leads to stress-test and refine their proposals against a synthetic audience. This process amplifies missing voices rarely heard in physical meetings and generates accessible materials that facilitate easier adoption and higher-quality engagement for subsequent human panels.

## 1 Introduction

Patient and Public Involvement and Engagement (PPIE) is widely recognised as a prerequisite for ethical and effective healthcare research (1). However, despite widespread recognition of its importance, the operational reality often falls short. Panels are frequently dominated by “frequent flyers” (2)—available, articulate individuals from majority demographics—while marginalised voices remain absent (3). This representational failure undermines the legitimacy and effectiveness of healthcare research designed to serve diverse populations. Furthermore, the materials presented to these panels—often dense, academic proposals—create cognitive barriers that prevent lay contributors from exercising genuine influence (4).

This paper introduces Panelyze™, a generative AI framework designed to bridge the gap between healthcare research design and public understanding. Unlike general-purpose Large Language Model (LLM) applications (5, 6), Panelyze employs a structured, multi-agent architecture to simulate diverse personas and generate the materials necessary for effective human engagement. We present the system's five-step workflow and introduce the Panelyze Score, a novel algorithmic metric derived from the UK Standards for Public Involvement (7). While the Score provides a quantifiable baseline, the system's primary innovation lies in its ability to surface qualitative “lived experience” insights *in silico*, allowing researchers to iteratively identify and resolve structural barriers in addition to engaging real patients.

Beyond its immediate utility in the co-production of PPIE panels, this framework addresses a critical dimension of technology adoption in the social sciences: AI for Inclusivity. While AI is often critiqued for perpetuating bias, this research explores its potential as a tool for public policy equity. By deliberately engineering synthetic agents to embody under-represented demographics, Panelyze leverages the capabilities of LLMs to shift the window of engagement, ensuring that digital health interventions are not designed solely by, and for, majority populations.

## 2 Materials and methods

### 2.1 System architecture

The Panelyze platform is built on a React and Node.js stack and is deployed as a web application. It features a bespoke prompt engineering framework developed specifically for PPIE use cases, enabling experimentation with a range of State-of-the-art (SOTA) Large Language Models (LLMs). The core simulation engine architecture is designed to ground LLM outputs in specific technical contexts, utilising text analytics to bridge the gap between unstructured clinical research protocols and structured, programmatically defined persona prompts.

Central to this architecture is the AIServiceFactory, a task-oriented orchestration layer that programmatically manages model selection and deployment. The AIServiceFactory acts as a traffic controller, routing specific linguistic and analytical tasks to a picklist of frontier models from providers including Anthropic, Google, and OpenAI. By abstracting the underlying API logic, the factory ensures the framework remains model-agnostic while ensuring that each stage of the research workflow is handled by the most capable agent.

**Figure 1:**
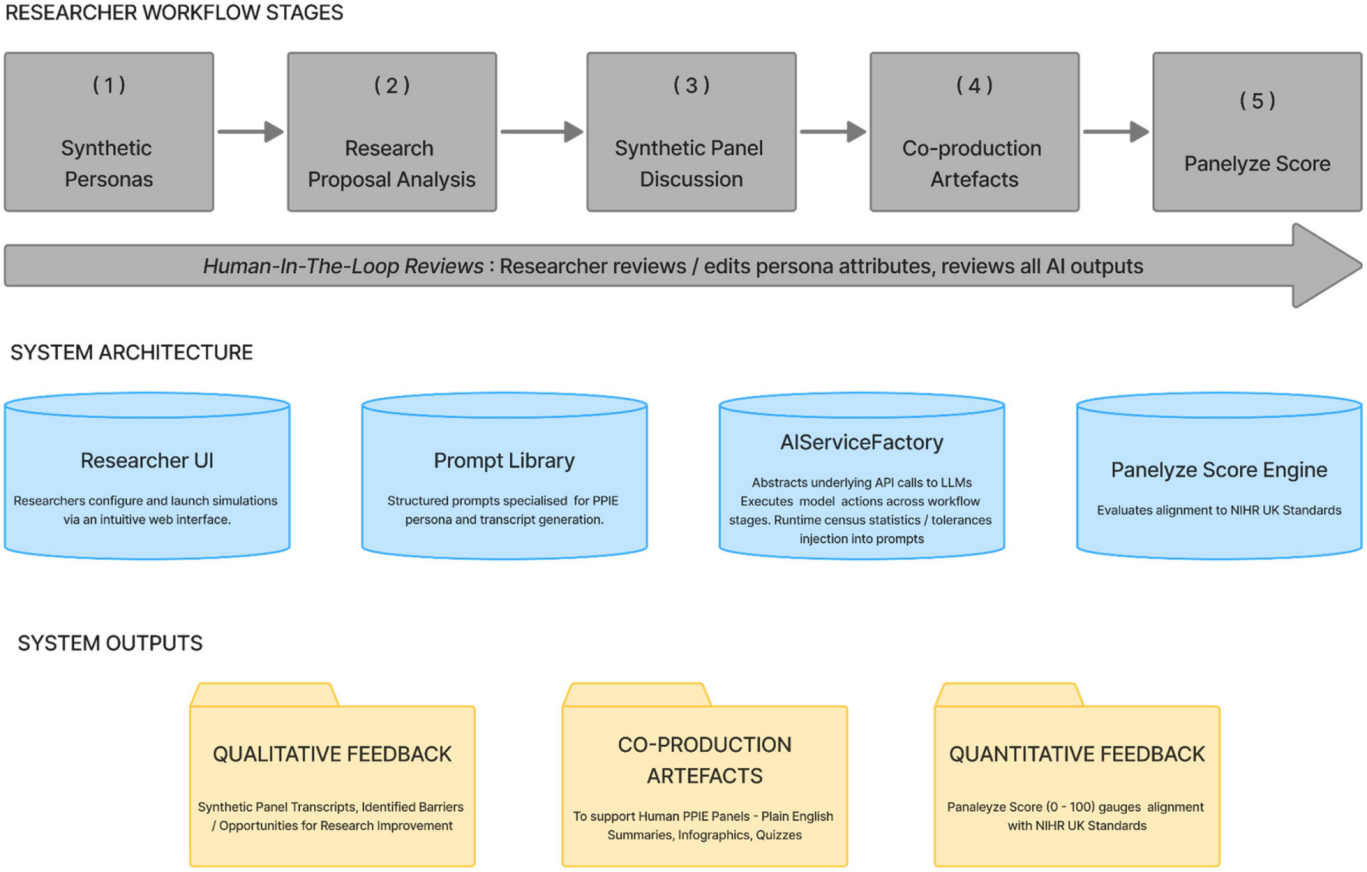
Panelyze high-level workflow, architecture and outputs. Displays the 5-step workflow, key architectural components, and qualitative and quantitative outputs from Panelyze.

In the default configuration, model assignment is optimised for distinct workflow requirements: Claude 4.5 is used for its high-context narrative capabilities to generate rich synthetic persona backstories and consistent panel transcripts; GPT-5 serves as the primary “Assessor Agent” to evaluate proposals against established NIHR standards (the Panelyze Score); and Gemini 2.5 Flash is employed for the generation of visual co-production artefacts and infographics. This orchestration facilitates a transparent, human-AI interaction loop, as detailed in Figure 1, ensuring that AI-generated insights are always subject to researcher oversight before implementation. In the current implementation, cross-validation of model outputs is not performed programmatically; rather, output fidelity is assured through the mandatory Human-in-the-Loop review phase described in Section 2.2. The introduction of an independent “Judge Model”—an AI agent tasked with auditing the fidelity and utility of generated personas and panel discussions—is identified as a near-term development priority and forms part of the platform's ongoing roadmap.

### 2.2 Generation of diverse synthetic personas and panels

To address PPIE representation gaps, the system leverages the inherent flexibility of software to programmatically model synthetic panels. Unlike real human panels, which are constrained by recruitment logistics, Panelyze allows researchers to configure a comprehensive set of persona attributes to reflect the target population. These configurable attributes span three categories: (1) the nine protected characteristics defined under the Equality Act 2010 (11); (2) socio-demographic variables including occupation, employment status and geographic location; and (3) narrative attributes comprising a free-text lived experience backstory that contextualises how the persona relates to the research domain.

This is achieved through a two-stage grounded generation process. First, to ensure statistical fidelity, the system performs runtime injection of aggregated census statistics [from Scotland Census 2022 (8), England & Wales Census 2021 (9), and US Census 2020 (10)] directly into the LLM system prompts as structured reference data. Rather than embedding raw census tables, the system extracts and formats the relevant distributional statistics for the target geography (e.g., age band proportions, ethnic group percentages, and occupational classification breakdowns) as a compact structured block within the prompt context. A tolerance check is then applied programmatically: the system calculates the attribute distribution across all generated personas and validates that each dimension falls within a predefined variance threshold (±10%) of the corresponding census baseline. If the distribution exceeds this tolerance, persona generation is re-triggered until the panel achieves acceptable demographic fidelity before proceeding to the simulation stage.

Second, the framework incorporates a critical “Human-in-the-Loop” phase before the simulation begins. Researchers have full flexibility to manually review and edit all generated persona attributes, including the protected characteristics, socio-demographic variables, and the AI-generated backstory. This editorial control allows investigators to precisely represent desired voices or specific lived experiences relevant to the research context—for example, deliberately over-sampling individuals with a particular disability status to stress-test accessibility provisions in a proposed protocol. Finally, researchers can select from predefined “Panel Profiles” (e.g., Efficient, Rich Narrative, Risk-Focused, or Innovation-Focused) which parameterise the LLM prompt to modulate narrative depth, discussion length, and the balance of response archetypes (sceptical, inquisitive, supportive) within the simulated discussion.

### 2.3 Document upload and semantic analysis

The Research Lead uploads the same documentation to Panelyze as that intended for the human panel. The system's semantic parsing engine processes this unstructured text, identifying key study parameters. It extracts core objectives, inclusion/exclusion criteria, and specific research questions to be addressed by the synthetic panel.

### 2.4 Synthetic discussion generation

Once the panel is generated and the proposal parsed, the platform accesses the prompt library and triggers a simulated 30-minute panel discussion (3,000–5,000 words). This is led by a synthetic PPIE Lead persona and follows a structured debate format: Opening, Initial Reactions, Deeper Exploration, Synthesis, and Closing. The simulation engine introduces a range of response types (sceptical, inquisitive, supportive) and variable contributions from synthetic personas to simulate authentic group dynamics, specifically designed to surface qualitative friction points.

### 2.5 Co-production artefact generation

Using the transcript from the simulated discussion and the original research proposal, the system creates co-production artefacts to bridge comprehension gaps. Examples include:

- Plain English Summaries: Calibrated to NHS standards for accessibility.
- Warm-Up Quizzes: Gamified tools for the human panel derived from friction points in the synthetic discussion.
- Patient Information Sheets: Addressing any concerns expressed by synthetic panels.
- Infographic Descriptions: Visual aids breaking down complex trial designs.

### 2.6 The “Panelyze Score” algorithm

To translate the qualitative output of a synthetic panel discussion into a reproducible quality metric, the platform computes a composite Panelyze Score (0–100). This metric is anchored to the six UK Standards for Public Involvement (7): Inclusive Opportunities, Working Together, Support & Learning, Communications, Impact, and Governance.

Rather than evaluating the submitted research proposal in isolation, a specialised “Assessor” agent analyses the synthetic panel's discourse to determine how well the proposal's provisions were reflected, challenged, or found absent in the simulated discussion. For each standard, the prompt provides explicit evaluation criteria and classifies what participants revealed in the transcript into three tiers: positive evidence (participants express explicit support backed by concrete proposal details); vague or mixed evidence (the standard is mentioned but lacks specifics or draws concern); and absent evidence (the proposal is silent on the standard or participants identify fundamental gaps). The rubric adopts a “sceptical default” principle, whereby silence on a standard is treated as a failure rather than a neutral omission. Each rationale is required to cite direct participant quotes from the transcript as supporting evidence, ensuring traceability between the qualitative discourse and the quantitative score.

This approach operates on the foundational assumption that census-grounded synthetic personas, when appropriately configured, constitute a reasonable proxy for the perspectives and concerns of individuals sharing the selected demographics and lived experiences—an assumption that warrants empirical validation in future comparative studies.

For each of the six standards, the model assigns an individual integer score (0–100) to every panel member, generating a participant-by-standard scoring matrix. Dimension-level scores are obtained by computing the panel mean for each standard, and the composite Panelyze Score is calculated as the unweighted mean across all six dimensions. This equal weighting is a deliberate design choice to prevent high performance in one domain (e.g., Communications) from masking structural deficits in another (e.g., Governance).

For practical interpretation, scores are mapped to five ordered quality categories: Critical (0–29), Poor (30–49), Moderate (50–69), Good (70–89), and Exceptional (90–100). Any dimension scoring below the Moderate threshold automatically triggers LLM-generated targeted improvement recommendations for the researcher. Ultimately, the Panelyze Score acts as a formative signal capturing what a census-calibrated patient cohort collectively articulates as aligned or deficient; it does not constitute a formal regulatory audit.

## 3 Results

To validate the operational utility of the Panelyze framework, we deployed the system to evaluate the CAPRIE-2 (Cardiovascular Primary Prevention Research) proposal, a large-scale cardiovascular trial with a ten-year duration focusing on long-term secondary prevention of coronary heart disease (12). Quantitative and qualitative outputs for the CAPRIE-2 validation study are summarised below, with the full dataset including simulated transcript and Panelyze Scores available as Supplementary Material.

### 3.1 Simulated qualitative inquiry: identification of barriers

While the system generates a numerical score, its primary utility was found in the semantic extraction of qualitative barriers. The synthetic panel acted as a “stress test” for the research proposal, identifying specific socio-economic friction points that would likely impede recruitment in the real world. As shown in Table 1, the system translated technical proposal requirements into human consequences. For example, regarding the study's longitudinal design, the synthetic panel moved beyond generic feedback to identify a “Critical” barrier regarding lost wages for working participants.

**Table 1:** Qualitative translation of proposal barriers by synthetic personas. Summarizes technical protocol elements alongside identified socio-economic barriers and verbatim feedback from synthetic personas.

Protocol element (CAPRIE-2)	Identified barrier (severity)	Synthetic persona “Verbatim” feedback
Visit schedule: 10-year follow-up requiring clinic visits.	Temporal/financial (critical)	"For working professionals..if it means missing pay, that's a big problem. Maybe financial help for lost earnings could be considered?"
Drug terminology: use of “Clopidogrel”.	Cognitive/familiarity (mixed sentiment)	"Clopidogrel sounds more technical and less familiar than Aspirin. Clear explanation..is crucial."
Dissemination: use of YouTube for patient info.	Digital Exclusion (Moderate)	"I worry about accessibility..Many of the individuals I support might not have regular access to the internet."

### 3.2 Concept testing and terminology validation

The system demonstrated the capacity to answer specific research questions posed by the investigators, validating strategic design choices before human implementation.

- Genetic Testing vs. Outcomes: When queried on the proposed decision to forego genetic testing in favour of observing outcomes across ethnicities, the panel consensus was Positive. The synthetic cohort validated the decision as “pragmatic” and “cost-effective”.
- Terminology Acceptance: Conversely, the system flagged potential friction regarding the drug name “Clopidogrel”. The panel returned a Mixed Sentiment result, noting that while the science was sound, the terminology was “less familiar than Aspirin” and required an explicit communication strategy.

### 3.3 Quantitative benchmarking via the Panelyze Score

**Figure 2:**
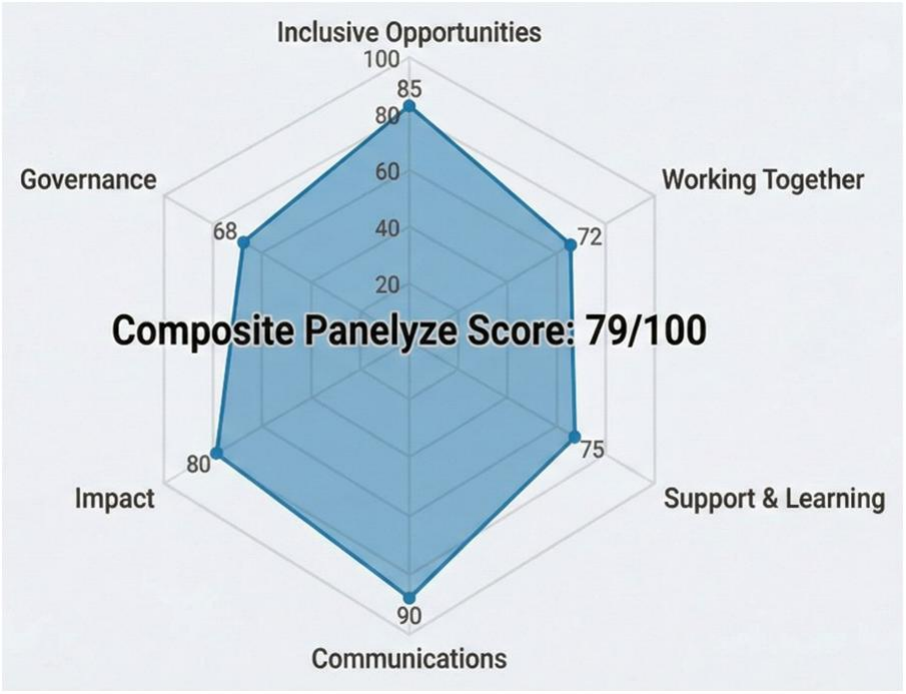
Panelyze Score radar chart for CAPRIE-2 research proposal. Displays the performance of the cardiovascular research proposal against the six UK Standards for Public Involvement.

The system assigned the CAPRIE-2 research proposal a composite Panelyze Score of 79/100, categorising the proposal as “Good”. As illustrated in Figure 2, the proposal performed unevenly across the UK Standards. It achieved its highest ratings in Communications (90/100) and Inclusive Opportunities (85/100), but performance dropped in Working Together (72/100) and Governance (68/100). Notably, the lower Governance score (68/100) was driven by the synthetic panel's observation that the current proposal lacked a dedicated PPIE advisory group.

## 4 Discussion

### 4.1 Operationalising quality: balancing metrics and narrative

A primary contribution of this work is the operationalisation of the UK Standards for Public Involvement into a dual-output framework. While the Panelyze Score provides a necessary benchmark, the primary value lies in surfacing specific, narrative friction points—such as the financial burden on working participants—that a numerical score alone cannot articulate.

### 4.2 Augmentation, not replacement

It is a fundamental tenet of this framework that Panelyze synthetic panels are designed to augment, not replace, human involvement. The system can serve as a preparatory filter, allowing research leads to practice and refine the research proposal against a synthetic audience in addition to consultation with existing public involvement panels. Crucially, the synthetic panels amplify voices that are often structurally missing from physical meetings.

### 4.3 Limitations and conclusion

This proof-of-concept explicitly acknowledges the risks inherent in generative AI, specifically hallucination and algorithmic bias. However, the context of PPIE offers a unique mitigation layer: unlike clinical diagnostics which require factual precision, PPIE is qualitative and sentiment-based. The researcher acts as a mandatory “human-in-the-loop” to vet synthetic outputs for plausibility. Regarding demographic-clinical coherence, the human review phase also serves to identify and correct any implausible mismatches between a persona's synthetic attributes and their simulated health experiences before the discussion proceeds.

Regarding bias, we do not claim to eliminate it, but rather to manage it. We trade the hidden structural bias of human recruitment (e.g., availability for daytime meetings) for the transparent, adjustable parameters of synthetic agents.

The current publication presents validation against a single case study (CAPRIE-2), selected as the only study for which full permissions to share outputs have been granted at this stage. The platform has been applied across approximately ten research proposals to date, and while those additional cases have informed iterative development, their outputs are not reported here pending investigator permissions. However, subsequent comparative work is underway to address this directly. Initial comparisons between Panelyze synthetic panel outputs and feedback from real PPIE members and facilitators have demonstrated significant convergence on key issues, suggesting the synthetic panels identify barriers and concerns that are genuinely recognised by human participants. Notably, both panels also surfaced distinct additional topics not raised by the other, suggesting that synthetic and human panels are complementary rather than substitutive—each adding independent value to the research lead. These preliminary findings further support the case for Panelyze as an augmentation tool and will inform the next phase of platform development.

Ultimately, Panelyze serves as a preparatory simulation environment to stress-test research protocols against diverse demographic perspectives. It utilises in silico modelling to identify barriers early, thereby optimising subsequent human engagement. Future studies are needed to quantify the system's impact on actual research protocols, trial conduct, and recruitment rates (13). Furthermore, the Panelyze Score should be interpreted as a formative benchmarking instrument rather than a psychometrically validated scale; the use of LLM-assigned integer scores as ordinal proxies is an acknowledged methodological limitation that future work should address through structured reliability testing.

In conclusion, Panelyze offers a scalable technological intervention to pressure-test healthcare research proposals and support human PPIE panels. By leveraging SOTA LLMs to simulate diverse perspectives, it facilitates inclusive research design and ensures healthcare innovation is grounded in the needs of the target population.

## Data Availability

The original contributions presented in the study are included in the article/Supplementary Material, further inquiries can be directed to the corresponding author.

## Supplementary material

Supplementary Data Sheet S1 Synthetic Panel Discussion Transcripts for the CAPRIE-2 Case Study.

Supplementary Data Sheet S2 Itemised Panelyze Score Breakdown against UK Standards for Public Involvement for the CAPRIE-2 Proposal.
