# Supplementary material for: Amplifying missing voices in healthcare research: an AI framework for co-production of PPIE
Source: Front Digit Health. 2026 Apr 7;8:1771729. doi: 10.3389/fdgth.2026.1771729 (PMC13096077; doi:10.3389/fdgth.2026.1771729)
Supplement: Supplementary Data Sheet S1 — Synthetic Panel Discussion Transcripts for the CAPRIE-2 Case Study. [file Datasheet1.pdf]

COMPREHENSIVE PPIE TRANSCRIPT

21 Nov 2025 ~23 min read CONFIDENTIAL

Comprehensive PPIE Panel Transcript – Appendix 2 Colin Berry_6March2024 v2.docx

Panelyze: Virtual PPIE Panel Transcript

Study

Study Title: CAPRIE-2: Clopidogrel versus aspirin monotherapy for secondary prevention: a comparative effectiveness, randomised trial (Plain English: In people who have coronary heart disease, is clopidogrel more beneficial than aspirin for preventing cardiovascular death? A randomised trial.) **PPIE Lead:** Sarah Davies **Duration:** 30 minutes

Panel Composition

- EM Eilidh McGregor (58, Retired Teacher) – CVD (Coronary Artery Disease)
- CM Callum MacLeod (45, IT Project Manager) – CVD (Hypertension)
- AK Anya Kowalska (67, Retired Nurse) – Good Health
- MK Mohammed Khan (32, Software Developer) – Good Health

- FC Fiona Campbell (72, Retired Librarian) - Good Health
- DS David Smith (29, Barista) - Good Health
- LO Liam O'Connell (37, Construction Worker) - Good Health
- FA Fatima Ahmed (25, University Student (Nursing)) - Good Health
- AB Andrew Bell (62, Retired Factory Worker) - CVD (Atrial Fibrillation)
- IS Irene Scott (49, Care Assistant) - Good Health
- KM Kwame Mensah (41, Taxi Driver) - Good Health

START of TRANSCRIPT

Opening (0:00–3:00)

SD Hello everyone, and a very warm welcome to our panel discussion today. My name is Sarah Davies, and I'm a Community Health Worker. I'll be facilitating our conversation this afternoon. We're here to discuss a research proposal called CAPRIE-2, which aims to compare two medications, clopidogrel and aspirin, for patients who have coronary heart disease. The plain English title is: "In people who have coronary heart disease, is clopidogrel more beneficial than aspirin for preventing cardiovascular death? A randomised trial."

This is a crucial time for us to get your valuable input as the research is currently at the initial design and funding application stage. The study plans to recruit a large number of participants, between 40,000 and 50,000, and will follow them for up to 10 years, starting

from June 2025 if funded by the British Heart Foundation and other international partners.

We have a fantastic group here today, representing a wide range of experiences and backgrounds. We have individuals with lived experience of cardiovascular disease, healthcare professionals, and members of the public with diverse occupations and ethnicities. Thank you all for being here and for sharing your time and expertise. We're aiming for a relaxed and open discussion, so please feel free to jump in and share your thoughts.

Let's do a quick round of introductions. Could each of you briefly introduce yourself, perhaps mentioning your location and any relevant background you'd like to share? We'll start with Eilidh.

EM Hi, I'm Eilidh McGregor, I'm 58 and I'm a retired primary school teacher from Glasgow. I have coronary artery disease after a heart attack five years ago, and I'm on medication and regularly attend the Queen Elizabeth University Hospital. I'm really keen to make sure cardiac care is as good as it can be.

CM Hello, I'm Callum MacLeod, 45, from Edinburgh. I work as an IT Project Manager and have managed hypertension for about ten years, which affects my cardiovascular health. I'm actively involved with my GP at Lothian Health Board. I want to contribute to making healthcare interventions accessible and effective.

AK Good afternoon. My name is Anya Kowalska. I'm 67 and live in Dundee. I'm a retired nurse, originally from Poland, and I worked within NHS Tayside. I'm in good health and have a strong interest in healthcare systems and patient experience.

MK Hi everyone, I’m Mohammed Khan, 32, based in Glasgow. I’m a software developer. I’m particularly interested in how technology can be better integrated into health research and care, especially for ethnic minority groups. I believe diverse voices are key to improving health outcomes.

FC Hello, I’m Fiona Campbell. I’m 72, a retired librarian from Aberdeen. I enjoy good health and I’m a keen reader of research. I want to help make health research accessible and understandable to the public.

DS Hi, I’m David Smith. I’m 29 and work as a barista in Perth. I’m keen to ensure research considers the needs of younger adults and those in service industry roles.

LO Afternoon. Liam O'Connell, 37, from Paisley. I work as a construction worker. I want to understand how health research can be made more accessible to people in manual trades.

FA Hello, I’m Fatima Ahmed, 25, a nursing student in Livingston. I’m passionate about patient-centred care and eager to contribute a fresh perspective from my studies and potential frontline experience.

AB Hi, I’m Andrew Bell, 62, from Hamilton. I’m a retired factory worker and I have atrial fibrillation, a type of cardiovascular disease. I receive care through NHS Lanarkshire and want to share my long-term patient perspective to improve cardiac care.

IS Hello, I’m Irene Scott, 49, from Kirkcaldy. I work as a care assistant and have direct experience supporting people with

various health needs. I'm motivated to ensure research reflects practical realities of care.

KM Good afternoon. Kwame Mensah, I'm 41, a taxi driver in Ayr. I'm from Ghana. I'm committed to contributing to research that benefits diverse communities, particularly addressing health disparities.

SD Thank you all so much for those introductions. It's wonderful to have such a diverse and experienced group here.

Initial Reactions (3:00–10:00)

SD Let's dive into the research proposal. As I mentioned, it's comparing clopidogrel to aspirin for people with coronary heart disease. Aspirin is something many people are familiar with, but clopidogrel is less common. My first question to the panel is: **Is the term 'Clopidogrel' off-putting to the public, given the familiarity of aspirin?** What are your initial thoughts on hearing that name?

EM Well, as someone with heart disease, I've heard of both. Aspirin is a household name, isn't it? Clopidogrel... it sounds a bit more technical, a bit more intimidating. If I hadn't had experience with it, I might wonder what it was. It doesn't roll off the tongue quite like aspirin.

CM I agree with Eilidh. Aspirin is something most people associate with pain relief or a simple preventative measure. Clopidogrel sounds like a more complex pharmaceutical. For someone who isn't actively managing a cardiovascular condition, it might raise

immediate questions or even a slight apprehension about its potency or potential side effects.

AK From a professional viewpoint, we used clopidogrel regularly in nursing. But I can see how the general public might find it a bit of a mouthful. Perhaps the plain English title helps, but the drug name itself could be a barrier to understanding or engagement if not explained very clearly.

MK I think it's less about the name itself and more about how it's presented. If it's just thrown out there without context, yes, it might seem off-putting. But if it's part of a well-explained research study that clearly outlines its purpose and benefits, then people might be more open to learning about it. The key is good communication.

FC I tend to agree with Mohammed. The scientific name itself is less of a concern for me than the clarity of the explanation. However, I do think there's a potential initial hurdle. If someone's only ever taken aspirin and suddenly they're being asked about clopidogrel, they might think, "Why change from what I know works, even if it's not perfect?"

DS I haven't really heard of clopidogrel before, to be honest. Aspirin, yeah, my nan takes it. But this other one, it sounds like it's for something serious. If I saw that name on a leaflet, I might skip over it thinking it's not for me, or too complicated.

LO Yeah, same here. Aspirin, everyone knows that. Clopidogrel... no clue. Sounds like something you'd need a doctor to explain properly. I'd want to know if it's better, or just different, and why.

AB I’ve been on clopidogrel myself, so it’s familiar to me. But I understand what everyone’s saying. It’s a much less familiar term than aspirin. I can see how it might put people off initially, especially if they’re already a bit anxious about their heart health or their medication.

IS As a care assistant, I hear patients talk about their medications. Aspirin is very common. Clopidogrel is used for some of my clients, but they often refer to it by its brand name, or just say "the other tablet." So, the generic name itself isn't widely recognised by the public.

KM I think it’s about building trust. If the research team can explain why clopidogrel might be beneficial, and assure people that it's being studied rigorously, then the name becomes less of an issue. But it definitely needs a clear, accessible explanation.

SD That’s a really consistent theme – clarity and explanation are key. It sounds like the name itself isn't inherently problematic, but it does require a strong communication strategy. Thank you for those initial thoughts.

Moving on to the study’s design. The research aims to include a diverse population, prioritising females and people from a broad range of ethnic backgrounds. The proposal mentions that for the CAPRIE study, which this builds on, 95% of participants were white and 72% were male, and the follow-up period was only two years. This new study is looking for 40,000–50,000 patients and will follow them for up to 10 years.

The researchers are proposing to answer questions about differences in effects by sex, ethnicity, and socio-economic deprivation. However, they are *not* proposing to perform expensive gene testing to assess clopidogrel response in different ethnicities. So, my next question is: **Is it reasonable in this large study not to perform expensive gene testing to assess clopidogrel response in different ethnicities, and instead answer the question on whether results truly differ by ethnicity?**

AK From a nursing perspective, I understand the desire for detailed biological information. Gene testing is becoming more common. However, for a study of this size and duration, the cost and logistical challenges of widespread genetic testing would be immense. It's a practical consideration. If the primary goal is to see if there are *observed* differences in outcomes across ethnic groups, then measuring those outcomes is the direct way to answer that, even without the genetic explanation.

MK I think this is a really important point. If the goal is to understand if clopidogrel works differently for different ethnicities in a real-world setting, then observing the outcomes in a diverse population is exactly what's needed. Gene testing would provide *why*, but not necessarily *if* there's a difference in effect. For a large-scale trial, focusing on observable outcomes is likely more feasible and directly answers the question of generalisability. We need to know if it works for people like me, regardless of the underlying genetic mechanism.

CM I see the argument. If the study can demonstrate that clopidogrel has a different effect or side effect profile in, say, South

Asian populations compared to white populations, that's valuable information in itself, even if we don't know the precise genetic reason. It flags a potential issue that might warrant further investigation later. The cost of widespread genetic testing in 50,000 people is astronomical.

EM As a patient, I'm not sure I'd understand why they'd need to test my genes. I'd just want to know if the medicine is safe and effective for me. If that means looking at how it works in different groups of people, and they can do that without making it too complicated or too expensive, then that sounds sensible. But I'd want to be sure they're really looking at diversity properly.

FC I agree with Anya and Mohammed. The question is about effectiveness and benefit across groups. If the study can recruit a representative sample and track outcomes, that's the most direct way to answer the question of whether results differ by ethnicity. Gene testing is a deeper dive that might be a follow-up study. For this large trial, focusing on the observable impact is pragmatic and crucial for real-world applicability.

DS It sounds like for the people taking part, they're not going to be poked and prodded with extra tests like gene stuff? That's good. I'm happy for them to look at whether it works differently for different people, as long as it doesn't mean more hassle for me.

LO Yeah, if it means more blood tests or something, that's a no from me. But if they're just looking at the results of the main treatment, then fine. As long as they're actually getting different people in the study in the first place.

AB I think it's about priorities. We've had issues with studies being too narrow in the past. If this study makes a real effort to include diverse ethnic groups and then *shows* that the outcomes are consistent, that's a massive win. It's more important that the drug is proven to work or not work across different people than to understand the exact genetic pathway for every single person. The cost of widespread genetic testing would likely make the study infeasible.

IS From my perspective, seeing the results across different ethnicities is vital. Many of the patients I care for come from diverse backgrounds, and it's essential that treatments are effective for everyone. If the researchers can demonstrate this through careful data collection on outcomes, that's a significant achievement without the added complexity and cost of genetic testing. It's about making sure the drug is safe and effective for all patient groups.

KM I strongly support this approach. The original CAPRIE study had significant limitations regarding ethnicity. To address that, this new study needs to actively recruit diverse populations and then analyse the outcomes. This directly answers the crucial question of whether the treatment is equitable. While genetic insights are valuable, they shouldn't be a prerequisite for assessing real-world effectiveness in different ethnic groups. The cost is a major factor for a study of this scale.

SD It seems there's a strong consensus that focusing on observable outcomes in a diverse population is the pragmatic and

appropriate approach for this large-scale study, given the cost and complexity of gene testing. Thank you for those insights.

Deeper Exploration (10:00–25:00)

SD Let's delve a bit deeper into how this research will reach and inform potential participants. The study is massive, 10 years long, and involves a large number of people. How will people understand what the study is about, the medications involved, and what's expected of them? The researchers are proposing to use YouTube videos as one method for providing study information. So, my third question is: **Are YouTube videos an acceptable method for providing study information, and does the panel have prior experience or recommendations for their development?**

MK I think YouTube is a brilliant idea for reaching a wide audience, especially younger demographics and those who are comfortable with digital media. It's accessible, can be visually engaging, and can be shared easily. However, it's crucial that these videos are made with accessibility in mind. Clear language, subtitles, perhaps even options for different languages. We need to ensure it's not just for tech-savvy people but for everyone.

AK As a former nurse, I'm a little cautious. While YouTube can be a useful tool, not everyone accesses information that way. Some people, particularly older individuals or those less familiar with technology, might not use YouTube at all. So, it should be *part* of a broader communication strategy, not the sole method. If they do use it, the videos need to be very clear, concise, and avoid jargon.

Perhaps animations could help explain complex concepts like clopidogrel versus aspirin.

FC I agree with Anya. YouTube is a good supplementary tool, but it can't be the only one. My concern is for those who might not be digitally connected or who prefer reading information on paper. For those who do use YouTube, the videos must be professional and engaging. I'd recommend ensuring they are reviewed by a PPI group *before* they are released, to check for clarity and any unintended messages.

EM I'm not on YouTube much myself. My grandchildren use it all the time, though. So, I can see the appeal. But I'd want to see a leaflet or a booklet as well, something I can keep and refer to. The video needs to be easy to follow. Maybe show real people talking about why they joined the study? That always makes it more real for me.

CM From an IT perspective, YouTube is a standard platform. It allows for updates too, which could be useful for a 10-year study. However, the quality of the video is paramount. Poorly made videos can be counterproductive. I think they should consider different lengths – a short overview, and then maybe more detailed videos on specific aspects like consent, or what to expect in terms of follow-up. And definitely consider subtitles and audio descriptions.

DS I use YouTube a lot for learning things. If there was a video explaining the study, I'd probably watch it. It's easier than reading a long document sometimes. But yeah, it needs to be clear. And maybe have a link to more info if I want to read up about it.

LO I'm not on YouTube much. Maybe if my mates told me about a good video. But I'd rather have a leaflet that I can read when I'm having a cuppa. If they make a video, it needs to be really, really simple. Like, what's the point of this drug? What will it do to me?

AB I've seen some good health information videos on YouTube. They can be quite effective. They need to be produced professionally, and crucially, they need to be accurate and balanced. I think it's a good idea, but it needs to be supplemented with other formats. A clear, concise written summary is essential.

IS While YouTube can be a modern way to disseminate information, I worry about accessibility for all patient groups. Many of the individuals I support might not have regular access to the internet or the devices to watch videos. A combination of formats is absolutely essential, including easy-to-read leaflets, potentially information sessions at local community centres, and clear communication from healthcare professionals.

KM YouTube is a powerful tool for outreach. For me, it's about ensuring the content resonates with diverse audiences. Can they feature people from different ethnic backgrounds in the videos? Can they explain the potential disparities in outcomes in a way that's informative and not alienating? For example, addressing the clopidogrel versus aspirin question for various communities. It's about making the information relevant and trustworthy for everyone. If they could include examples of how the medication has helped different people, that would be very impactful.

SD That's a very clear message: YouTube can be a valuable tool, but it must be part of a multi-channel approach, ensuring accessibility for everyone, regardless of their digital literacy or preferences. Professional production and clear, inclusive content are key.

Let's circle back to the inclusion of diverse populations. The proposal mentions prioritising females and people from a broad range of ethnic backgrounds and socio-economic deprivation. This is great. However, with a 10-year study, recruitment and retention can be challenging. What are the potential practical barriers to participation for different groups, and how can the study team overcome them?

EM For people like me, with existing heart conditions, it's about appointments. If the study requires lots of extra visits that are hard to get to, especially if you're not near a major hospital, it's a barrier. And if the medication has side effects, those need to be managed well.

CM Time commitment is a big one. For working professionals, taking time off for appointments can be difficult. Also, understanding the consent process. For something that lasts 10 years, that's a significant commitment. Clear, straightforward language in the consent forms, and perhaps a chance to discuss it with a study team member multiple times, would be beneficial.

MK For ethnic minority groups, trust in the research system can sometimes be an issue, stemming from historical experiences or lack of representation. Actively engaging community leaders or

organisations, and having researchers from diverse backgrounds, can help build that trust. Also, ensuring that information is available in languages that people understand is critical.

AK From a nursing perspective, I'd think about the practicalities for older patients or those with mobility issues. Transport to appointments is a major concern. Could there be options for virtual check-ins where appropriate? Or reimbursement for travel? And for those with cognitive impairment, ensuring a carer or family member is involved in the consent process would be important.

FC Accessibility of information is key, as we've discussed. But also, ensuring people understand the *value* of their participation. Why is this study important? How will it make a difference? Clearly articulating the potential impact on future patients, and how their contribution is vital, can motivate people to join and stay involved.

DS For younger people or people in jobs where you're on your feet all day, like mine, it's hard to get away for appointments. If it means missing pay, that's a big problem. Maybe financial help for travel or lost earnings could be considered?

LO For manual workers, health can be tied to being able to work. If the study involves something that might temporarily impact their ability to do their job, or requires long recovery periods, that's going to be a barrier. Also, making sure the language used is down-to-earth and not overly academic.

AB For long-term studies, maintaining engagement is tough. People's circumstances change. It's important for the research team to have good communication channels, to keep participants

informed about the study's progress, and to remind them why their part is important. A regular newsletter, perhaps, or updates on findings.

IS Many of my clients are on fixed incomes or have limited transport. Practical support, like travel expenses, or even offering appointments at more accessible local clinics rather than just major hospitals, could make a big difference. Also, ensuring that the study team is patient, understanding, and respectful of people's time and commitments is crucial.

KM For ethnic minority groups, as Mohammed mentioned, trust is key. This can be built by having diverse research staff, providing information in multiple languages, and working with community organisations to reach people. It's about demonstrating that this research is for *them*, not just for the academic community. Also, addressing any cultural beliefs or concerns they might have about the medications or the research process.

SD Excellent points, everyone. The themes of accessibility, financial considerations, building trust, and ongoing engagement are clearly critical for successful recruitment and retention, especially in a diverse population over a long period.

Synthesis (25:00–29:00)

SD We've covered a lot of ground in our discussion today. To quickly summarise some of the key takeaways:

Regarding the term 'Clopidogrel', while it's less familiar than aspirin, the panel agreed that with clear, accessible explanations and a strong communication strategy, it's not an insurmountable barrier. The focus should be on explaining *why* it's being studied and its potential benefits.

On the question of gene testing versus observing outcomes for ethnicity, there's a strong consensus that for a large-scale study like this, focusing on observed outcomes in a diverse population is the most pragmatic and effective approach. This directly answers the question of whether results differ by ethnicity in real-world settings, and the cost of gene testing makes it unfeasible for this study.

And regarding the use of YouTube videos, the panel viewed them as a valuable supplementary tool for information dissemination, particularly for reaching certain demographics. However, it's absolutely essential that they are part of a broader communication strategy that includes more traditional formats like leaflets and community outreach, ensuring accessibility for all.

Finally, we discussed potential barriers to participation. These are diverse and include practical issues like travel and time commitments, financial considerations, the importance of building trust within ethnic minority communities, and the need for ongoing engagement and clear communication throughout the 10-year study duration.

Does anyone have any final thoughts or concrete suggestions they'd like to add to this synthesis?

AK I think the researchers should really consider a dedicated PPI advisory group for this study, who can help shape the communication materials and advise on recruitment strategies.

MK And ensure that the people developing the YouTube videos and other materials truly understand the target audiences. Perhaps involve people from those communities in the creation process.

EM And keep us updated! It's good to know what's happening with the research we've given feedback on.

SD Those are excellent suggestions, Anya, Mohammed, and Eilidh. Having a dedicated PPI advisory group and involving diverse voices in material development are crucial. And keeping participants informed is vital for engagement.

Closing (29:00–30:00)

SD We are just about out of time. I want to express my sincere gratitude to each and every one of you for your thoughtful contributions today. Your insights are invaluable and will undoubtedly help the research team to refine their approach to making this important study as inclusive, accessible, and impactful as possible.

We will collate all the feedback from this discussion and share it with the research team. Your input is a critical part of ensuring that health research truly serves the needs of the communities it aims to benefit.

Thank you again for your time, your expertise, and your passion for improving healthcare.

END of TRANSCRIPT

Key Insights

- **Drug Name Familiarity:** While 'Clopidogrel' is less familiar than 'Aspirin', clear and accessible communication, focusing on the "why" and potential benefits, can overcome this initial barrier.
- **Ethnicity and Gene Testing:** For a large-scale study, observing outcomes in a diverse population is more pragmatic and cost-effective than extensive gene testing to determine ethnicity-based differences in drug response. This directly addresses the research question of real-world effectiveness across groups.
- **Communication Channels:** YouTube is a valuable supplementary tool for information dissemination, but it must be part of a multi-channel strategy that includes traditional formats (leaflets, community outreach) to ensure broad accessibility, especially for older adults or those with lower digital literacy. Professional, clear, and inclusive content is paramount.
- **Barriers to Participation:** Key barriers include practicalities like travel, time commitment, and financial implications. Building trust, especially within ethnic minority communities through representation and culturally sensitive communication, is vital. Ongoing engagement and clear communication throughout the long-term study are essential for recruitment and retention.
- **PPI Involvement:** Establishing dedicated PPI advisory groups and involving diverse community members in the development of communication materials are recommended for effective research engagement.
