# Supplementary material for: Amplifying missing voices in healthcare research: an AI framework for co-production of PPIE
Source: Front Digit Health. 2026 Apr 7;8:1771729. doi: 10.3389/fdgth.2026.1771729 (PMC13096077; doi:10.3389/fdgth.2026.1771729)
Supplement: Supplementary Data Sheet S2 — Itemised Panelyze Score Breakdown against UK Standards for Public Involvement for the CAPRIE-2 Proposal. [file Datasheet2.pdf]

PANELYZE SCORE REPORT 10 Dec 2025 ~12 min read CONFIDENTIAL

# Panelyze Score Report – 10/12/2025

## Panelyze Score Report

**Session:** cc4cfa1a... **Generated:** Wednesday, 10 December 2025 at 22:16 **Panel Size:** 12 participants **Document:** Research Document **Framework:** UK Standards 2019

## Overall Score: 79% (Good)

## NIHR 6 Standards Assessment

| Dimension | Score | Rating |
|---|---|---|
| Inclusive Opportunities | 72% | Good |
| Working Together | 85% | Good |
| Support & Learning | 78% | Good |
| Communications | 90% | Exception |
| Impact | 82% | Good |
| Governance | 68% | Moderate |

**Score Categories:**

- **90-100%:** Exceptional - Outstanding public involvement practice
- **70-89%:** Good - Solid public involvement with minor improvements possible
- **50-69%:** Moderate - Acceptable but significant room for improvement
- **30-49%:** Poor - Substantial improvements needed
- **0-29%:** Critical - Urgent attention required

## Dimension Analysis

### 1. Inclusive Opportunities (72% - Good)

- **Weight:** 16.67% of overall score

- **NIHR Definition:**

> *We offer public involvement opportunities that are accessible and that reach people and communities according to research needs.*

- **Panel Assessment:** The proposal shows intent for inclusivity by aiming for diverse recruitment, but participants highlighted significant barriers related to travel, time, financial implications, and digital access, suggesting the proposal needs more concrete strategies to overcome these for certain groups.

- **Individual Participant Scores:**

| Participant | Score |
|---|---|
| Eilidh McGregor | 70% |

| | |
|---|---|
| Callum MacLeod | 75% |
| Anya Kowalska | 70% |
| Mohammed Khan | 70% |
| Fiona Campbell | 75% |
| David Smith | 70% |
| Sarah Davies | 70% |
| Liam O'Connell | 70% |
| Fatima Ahmed | 75% |
| Andrew Bell | 70% |
| Irene Scott | 75% |
| Kwame Mensah | 70% |

## 2. Working Together (85% - Good)

**Weight:** 16.67% of overall score

**NIHR Definition:**

> *We work together in a way that values all contributions, and that builds and sustains mutually respectful and productive relationships.*

**Panel Assessment:** The panel's extensive discussion and detailed feedback on the proposal's design and communication suggest a strong potential for collaborative working. Participants actively offered suggestions for improvement, indicating a willingness to engage as partners, though the proposal itself doesn't explicitly detail co-production mechanisms.

### Individual Participant Scores:

| Participant | Score |
|---|---|
| Eilidh McGregor | 85% |
| Callum MacLeod | 85% |
| Anya Kowalska | 85% |
| Mohammed Khan | 85% |
| Fiona Campbell | 85% |
| David Smith | 85% |
| Sarah Davies | 85% |
| Liam O'Connell | 85% |
| Fatima Ahmed | 85% |
| Andrew Bell | 85% |
| Irene Scott | 85% |
| Kwame Mensah | 85% |

## 3. Support & Learning (78% - Good)

**Weight:** 16.67% of overall score

**NIHR Definition:**

> *We offer and promote support and learning that builds confidence and skills for public involvement in research.*

**Panel Assessment:** While the proposal doesn't explicitly mention formal support or learning opportunities for participants, the panel's discussion on clear communication and the need for accessible

information implies an understanding of what's required. The suggestion for a dedicated PPI advisory group indicates a potential for future learning structures.

**Individual Participant Scores:**

| Participant | Score |
|---|---|
| Eilidh McGregor | 75% |
| Callum MacLeod | 80% |
| Anya Kowalska | 75% |
| Mohammed Khan | 75% |
| Fiona Campbell | 80% |
| David Smith | 75% |
| Sarah Davies | 75% |
| Liam O'Connell | 75% |
| Fatima Ahmed | 80% |
| Andrew Bell | 75% |
| Irene Scott | 80% |
| Kwame Mensah | 75% |

## 4. Communications (90% - Exceptional)

**Weight:** 16.67% of overall score

**NIHR Definition:**

> *We use plain language for timely, two-way and targeted communications, as standard.*

**Panel Assessment:** The panel's consensus on the need for clear, accessible communication, particularly regarding the drug name and study purpose, and their positive reception of YouTube as a supplementary tool (with caveats for accessibility) indicates that the proposal's communication strategy, if implemented thoughtfully, could be very effective. The emphasis on plain language and multi-channel approaches is a strength.

**Individual Participant Scores:**

| Participant | Score |
|---|---|
| Eilidh McGregor | 90% |
| Callum MacLeod | 90% |
| Anya Kowalska | 90% |
| Mohammed Khan | 90% |
| Fiona Campbell | 90% |
| David Smith | 90% |
| Sarah Davies | 90% |
| Liam O'Connell | 90% |
| Fatima Ahmed | 90% |
| Andrew Bell | 90% |
| Irene Scott | 90% |
| Kwame Mensah | 90% |

## 5. Impact (82% - Good)

**Weight:** 16.67% of overall score

### NIHR Definition:

> *We seek improvement by identifying and sharing the difference that public involvement makes to research.*

**Panel Assessment:** The panel clearly understood and articulated the potential impact of the research, particularly regarding addressing ethnic disparities. Their detailed feedback on recruitment and communication aims to maximize the study's real-world impact and ensure findings are relevant to diverse populations.

### Individual Participant Scores:

| Participant | Score |
| --- | --- |
| Eilidh McGregor | 80% |
| Callum MacLeod | 85% |
| Anya Kowalska | 80% |
| Mohammed Khan | 80% |
| Fiona Campbell | 85% |
| David Smith | 80% |
| Sarah Davies | 80% |
| Liam O'Connell | 80% |
| Fatima Ahmed | 85% |
| Andrew Bell | 80% |
| Irene Scott | 85% |
| Kwame Mensah | 80% |

## 6. Governance (68% - Moderate)

**Weight:** 16.67% of overall score

**NIHR Definition:**

> *We involve the public in our governance and leadership so that our decisions promote and protect the public interest.*

**Panel Assessment:** The proposal's governance is moderately addressed. While the scale of the study implies significant resources, the panel's discussion on the need for a dedicated PPI advisory group and involvement in material development suggests that current governance might not fully integrate public input at the highest decision-making levels, and resource allocation for PPIE needs explicit consideration.

**Individual Participant Scores:**

| Participant | Score |
|---|---|
| Eilidh McGregor | 65% |
| Callum MacLeod | 70% |
| Anya Kowalska | 65% |
| Mohammed Khan | 65% |
| Fiona Campbell | 70% |
| David Smith | 65% |
| Sarah Davies | 65% |
| Liam O'Connell | 65% |
| Fatima Ahmed | 70% |

| | |
|---|---|
| Andrew Bell | 65% |
| Irene Scott | 70% |
| Kwame Mensah | 65% |

## Barriers Identified

### 1. temporal - Severity: critical

**Description:** Time commitment for appointments and potential need to take time off work, especially for those in manual labour or service industries.

> *"For working professionals, taking time off for appointments can be difficult."*

**Recommendation:** Offer flexible appointment scheduling, consider virtual check-ins where feasible, and explore reimbursement for lost earnings/travel.

### 2. financial - Severity: critical

**Description:** Financial implications of participation, including potential lost earnings and costs associated with travel or childcare.

> *"If it means missing pay, that's a big problem. Maybe financial help for travel or lost earnings could be considered?"*

**Recommendation:** Provide clear information on compensation for time and expenses, and offer allowances for travel and childcare.

## 3. physical - Severity: major

**Description:** Physical accessibility of study sites and transport to appointments, particularly for older individuals or those with mobility issues.

> *"Transport to appointments is a major concern. Could there be options for virtual check-ins where appropriate? Or reimbursement for travel?"*

**Recommendation:** Ensure study sites are physically accessible and provide comprehensive travel support, including options for local clinics or virtual consultations.

## 4. cultural - Severity: major

**Description:** Building trust within ethnic minority communities, stemming from historical experiences or lack of representation in research.

> *"For ethnic minority groups, trust in the research system can sometimes be an issue, stemming from historical experiences or lack of representation."*

**Recommendation:** Involve diverse researchers, partner with community leaders/organisations, and ensure information is available in multiple languages.

## 5. cognitive - Severity: moderate

**Description:** Understanding complex information and the consent process for a long-term study, especially for individuals with cognitive impairment.

> *"And for those with cognitive impairment, ensuring a carer or family member is involved in the consent process would be important."*

**Recommendation:** Provide consent information in multiple formats, allow ample time for discussion, and involve carers/family members where appropriate.

## 6. digital - Severity: moderate

**Description:** Digital literacy and access to technology for information dissemination, particularly for older adults or those with limited internet access.

> *"While YouTube can be a modern way to disseminate information, I worry about accessibility for all patient groups. Many of the individuals I support might not have regular access to the internet or the devices to watch videos."*

**Recommendation:** Ensure a multi-channel communication approach that includes non-digital formats like leaflets and community outreach.

## Enhancement Opportunities

## 1. participant_engagement - Criticality: high

**Description:** Establish a dedicated PPI advisory group to guide material development and recruitment strategies.

> *"I think the researchers should really consider a dedicated PPI advisory group for this study, who can help shape the communication materials and advise on recruitment strategies."*

**Implementation:** Formally establish a PPI advisory group with diverse representation and provide them with clear terms of reference and support.

## 2. communication - Criticality: high

**Description:** Involve diverse community members in the creation and review of communication materials, including YouTube videos.

> *"And ensure that the people developing the YouTube videos and other materials truly understand the target audiences. Perhaps involve people from those communities in the creation process."*

**Implementation:** Co-design and pilot all communication materials with representatives from target diverse groups.

## 3. research_design - Criticality: medium

**Description:** Maintain ongoing communication with participants about study progress and findings to foster engagement over the 10-year duration.

> *"And keep us updated! It's good to know what's happening with the research we've given feedback on."*

**Implementation:** Develop a clear communication plan for participants, including regular newsletters, study updates, and sharing of findings.

## 4. accessibility_enhancement - Criticality: medium

**Description:** Consider offering information and support in multiple languages to ensure inclusivity for diverse ethnic backgrounds.

> *"Also, ensuring that information is available in languages that people understand is critical."*

**Implementation:** Translate key study documents and communication materials into relevant languages based on target recruitment demographics.

## 5. innovation - Criticality: medium

**Description:** Utilize peer recruitment networks or patient advocacy groups to build trust and reach specific communities.

> *"Actively engaging community leaders or organisations, and having researchers from diverse backgrounds, can help build that trust."*

**Implementation:** Partner with established patient advocacy groups and community organizations for recruitment and outreach.

## 6. process_improvement - Criticality: medium

**Description:** Provide clear, simple explanations of the study's purpose, medications, and what is expected of participants, especially for those less familiar with research.

> *"Yeah, if it means more blood tests or something, that’s a no from me. But if they’re just looking at the results of the main treatment, then fine. As long as they're actually getting different people in the study in the first place."*

**Implementation:** Develop a tiered information approach: a concise overview, followed by more detailed explanations available in various formats.

## Research Questions & Answers

### Q1: Is the term ‘Clopidogrel’ off-putting to the public, given the familiarity of aspirin?

**Panel Sentiment:** ⚖️ Mixed

**Summary:** The panel generally agreed that 'Clopidogrel' is less familiar and sounds more technical than 'Aspirin'. However, they believe this is not an insurmountable barrier if the term is clearly explained within the context of the study's purpose and potential benefits, emphasizing the need for strong communication strategies.

**Key Discussion Points:**

Clopidogrel sounds more technical and less familiar than Aspirin.
Clear explanation of its purpose and benefits is crucial.
Communication strategy is key to overcoming potential apprehension.
Not inherently off-putting, but requires careful handling.

## Q2: Is it reasonable in this large study not to perform expensive gene testing to assess clopidogrel response in different ethnicities, and instead answer the question on whether results truly differ by ethnicity?

**Panel Sentiment:** ✅ Positive

**Summary:** There was a strong consensus that it is reasonable and pragmatic for this large-scale study not to perform expensive gene testing. The panel agreed that focusing on observable outcomes in a diverse population is the most effective way to answer whether results truly differ by ethnicity, given the cost and logistical challenges of genetic testing.

**Key Discussion Points:**

Focusing on observable outcomes is pragmatic and cost-effective for a large study.
Directly answers the question of whether results differ by ethnicity in real-world settings.
Gene testing is too expensive and logistically challenging for this scale.
Prioritizes real-world effectiveness over underlying genetic mechanisms for this study.

## Q3: Are YouTube videos an acceptable method for providing study information, and does the panel have prior experience or recommendations for their development?

**Panel Sentiment:** ⚖️ Mixed

**Summary:** The panel viewed YouTube videos as an acceptable and valuable supplementary tool for disseminating study information, particularly for reaching certain demographics. However, they stressed that it must be part of a broader communication strategy that includes traditional formats to ensure accessibility for all, and emphasized the need for professional, clear, and inclusive content.

**Key Discussion Points:**

YouTube is a good supplementary tool, especially for digital natives.
Must be part of a multi-channel communication strategy, not the sole method.
Accessibility for non-digital users (e.g., older adults) is critical.
Videos need to be professional, clear, concise, and inclusive (e.g., subtitles, diverse representation).

*Generated by Panelyze Healthcare PPIE Platform Based on UK Standards 2019*
